# Phosphorylation of PBK at Thr9 by CDK5 correlates with invasion of prolactinomas

**DOI:** 10.1111/cns.14629

**Published:** 2024-02-16

**Authors:** Qiuyue Fang, Changxiaofeng Liu, Ding Nie, Jing Guo, Weiyan Xie, Yazhuo Zhang

**Affiliations:** ^1^ Beijing Neurosurgical Institute Capital Medical University Beijing China; ^2^ Department of Neurosurgery, Beijing Tiantan Hospital Capital Medical University Beijing China; ^3^ Beijing Institute for Brain Disorders Brain Tumor Center, China National Clinical Research Center for Neurological Diseases Key Laboratory of Central Nervous System Injury Research Beijing China

**Keywords:** CDK5, EMT, PBK, phosphorylation, prolactinoma

## Abstract

**Context:**

Prolactinomas are the most prevalent functional pituitary neuroendocrine tumors (PitNETs), and they are invasive to surrounding anatomic structures. The detailed mechanisms of invasion are not yet clear.

**Objective:**

We explored the role of PBK phosphorylation in the proliferation and invasion of prolactinomas and its possible mechanism.

**Results:**

We report that PBK directly binds to and is phosphorylated at Thr9 by cyclin‐dependent kinase 5 (CDK5), which promotes GH3 cell EMT progression and proliferation. Phosphorylation of PBK at Thr9 (pPBK‐T9) by CDK5 enhances the stability of PBK. p38 is one of the downstream targets of PBK, and its phosphorylation is reduced as pPBK‐T9 increases in vivo and in vitro. Furthermore, we found that pPBK‐T9 is highly expressed in invasive PitNETs and was significantly correlated with invasion by univariate and multivariate analyses.

**Conclusions:**

Phosphorylation of PBK at Thr9 by CDK5 promotes cell proliferation and EMT progression in prolactinomas.

## Graphical abstract

The level of pPBK‐T9 is highly expressed in invasive PitNETs, CDK5 could promote the phosphorylation of PBK‐T9 through the p38 MAPK signaling pathway to promote cell proliferation and EMT progression. we revealed that this signaling axis in PitNETs, CDK5/PBK/p38 MAPK, promoted the tumorigenesis of PitNETs.

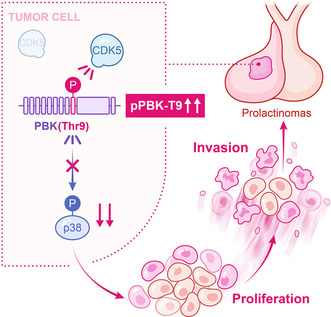

## 1 INTRODUCTION

Pituitary neuroendocrine tumors (PitNETs) are one of the most common intracranial tumors, and prolactinomas account for 40%–60% of all kinds of PitNETs. The clinical symptoms include galactorrhea, hyperprolactinemia, and infertility in women as well as mammoplasty and impotence in men.^1^, ^2^ Invasion prolactinomas are often more aggressive in invading the neighboring cavernous sinuses, which often grow rapidly and have a large size and poor prognosis.^3^, ^4^ The clinical diagnosis and treatment of prolactinomas that infiltrate the cavernous sinus present numerous complexities and obstacles, including challenges in preoperative classification, imprecise prognostic predictions, incomplete tumor excisions, postoperative recurrences, and resistance to pharmaceutical interventions. PitNETs can often be treated by surgery alone, or, as in the case of prolactinomas, may be managed medically.^5^ Dopamine agonists, including bromocriptine and cabergoline, are the major first‐line therapeutic drugs.^6^ Therefore, it is urgent to identify the key genes and pathways that play an important role in the occurrence and development of prolactinomas to help clinicians provide an accurate diagnosis and prognosis and to develop new therapeutic targets.

PDZ‐binding kinase (PBK), also known as T‐LAK cell‐originated protein kinase (TOPK), is highly expressed in a variety of human tumors.^7^, ^8^, ^9^, ^10^ As a member of the novel MEK3/6‐related mitogen‐activated protein kinase–kinase (MAPKK) family,^8^, ^11^, ^12^, ^13^, ^14^ it can phosphorylate AP‐1 (c‐Jun), ERK, JNK1, and MSL1 to affect cell proliferation, drug resistance, and poor prognosis. Research on the function of PBK as an oncogene is increasing, and its inhibitors are receiving increasing attention. Previous studies have demonstrated the essential roles of PBK in prolactinomas, and the TOPK inhibitor HI‐TOPK‐032 could suppress the proliferation and migration of prolactinomas by mediating p38 MAPK.^15^ CDK5 is a serine/threonine kinase that belongs to the mitotic cyclin‐dependent kinase family. Abnormal activation of CDK5 is closely related to the occurrence of a variety of tumors, including pancreatic cancer and prostate cancer.^16^, ^17^, ^18^ Our group has conducted research on the function of CDK5 for many years and has shown that CDK5 is involved in regulating several cellular functions in PitNETs. We have previously reported that CDK5 phosphorylates KDR (Ser‐229) and that Pit‐1 (Ser126) can directly promote tumor cell proliferation and invasion.^19^ As a serine/threonine kinase. Whether CDK5 can phosphorylate PBK has not been reported.

In this study, we identified Thr9 as a novel site on PBK that can be phosphorylated by CDK5. The phosphorylation of PBK at Thr9 by CDK5 increases PBK stability and promotes the tumorigenesis of PitNETs. We also verified that PBK is activated at Thr9 during the development of invasive prolactinomas and revealed a possible molecular mechanism in invasive PitNETs.

## 2 MATERIALS AND METHODS

### 2.1 Cell culture

Rat pituitary cells (GH3) were procured from the China Infrastructure of Cell Line Resources and cultivated in 35 mm dishes. The cells were nurtured in ATCC‐formulated F‐12 K medium (Invitrogen), supplemented with 2.5% fetal bovine serum (Gibco) and 15% horse serum (Gibco), within a controlled environment of a 37°C incubator with a humidified atmosphere consisting of 95% air and 5% CO2. The culture medium was refreshed every alternate day.

### 2.2 Plasmid construction and CDK5 inhibitor

The CDK5 siRNA (SR507441) construct was procured from OriGene Technologies located in Rockville, MD, USA. Mutant PBK(FLAG‐PBK‐T9F), PBK, and CDK5 were generated by Syngentech in Beijing, China. The authenticity of all constructs was verified through DNA sequencing conducted by Shanghai Shenggong Bio in China. Roscovitine was acquired from Sigma‐Aldrich (R7772) in St. Louis, MO, USA.

### 2.3 Cell counting kit‐8 (CCK‐8) assay

Cells were initially seeded in 96‐well plates at a density of 1 × 104 cells per well in 100 μL of cell culture medium for 24 h. Subsequently, the cells were transiently transfected with the specified plasmids and short interfering RNA. To assess cell viability, the CCK‐8 assay kit (Dojindo, Japan) was employed. After incubation, 10 μL of CCK‐8 solution was introduced to each well of the 96‐well plate and cultured for 3 h within an incubator. The optical density was then measured at 450 nm, and a proliferation curve was generated based on the relationship between time and absorbance.

### 2.4 EdU

The EdU assay was employed to evaluate the proportion of cells undergoing DNA replication, indicating their proliferation status. This assessment was conducted using an EdU detection kit (RiboBio). The EdU incorporation rate was determined by dividing the number of cells incorporating EdU by the number of cells stained with Hoechst 33342. A minimum of 500 cells were counted for each experimental group.

### 2.5 Wound healing assays

Cells were seeded onto Imagelock 96‐well plates (Essen Bioscience). The IncuCyte® Wound Maker (Essen Bioscience) was employed to create standardized wounds in a monolayer of confluent cells. Phase contrast imaging was conducted at 12‐h intervals until the 72‐h mark.

### 2.6 LC–MS/MS Analysis

The analysis of tryptic peptides was performed using a quadrupole Orbitrap mass spectrometer (Orbitrap Exploris™ 480, Thermo Fisher Scientific) coupled to an EASY nLC 1200 ultra‐high pressure system (Thermo Fisher Scientific) through a nano‐electrospray ion source.

### 2.7 The identification and quantitation of protein

The MaxQuant suite was employed to analyze all RAW files. The MS2 spectra were queried against the UniProtKB human proteome database, which encompasses both Swiss‐Prot and TrEMBL human reference protein sequences. This database consisted of 20,373 target sequences that were downloaded on 17 March 2022. The Sequest HT search engine was utilized, with the following parameters specified: complete tryptic specificity, allowance for a maximum of two missed cleavages, a minimum peptide length of 6, fixed carbamidomethylation of cysteine residues (+57.02146 Da), variable modifications accounting for oxidation of methionine residues (+15.99492 Da), a precursor mass tolerance of 15 ppm, and a fragment mass tolerance of 0.02 Da for MS2 spectra collected in the Orbitrap. Peptide spectral matches and peptides were filtered using Percolator to achieve a false discovery rate (FDR) of less than 1%.

### 2.8 Western blot analysis and antibodies

Protein samples underwent separation on 8%–10% Bis‐Tris SDS‐PAGE gels and were subsequently transferred onto polyvinylidene fluoride membranes (Merk). The primary antibodies were diluted in TBST solution supplemented with 1% bovine serum albumin (BSA) and were incubated with the membranes overnight at a temperature of 4°C. The visualization of immunoreactive bands was achieved through the utilization of chemiluminescence. The following antibodies were used in this study: CDK5 (ab40773, 1/200), pPBK‐T9 (ab184953, 1/1000), Snail (ab216347, 1/1000), N‐cadherin (ab18203, 1/1000), PBK (ab236872, 1/1000), E‐cadherin (ab76319, 1/1000) obtained from Abcam. FLAG (YN5598 1/5000), GFP (YM3009 1/5000), and HA (MY3003 1/5000) were obtained from Immunoway. JNK (9252P 1/1000), p‐JNK (4668S 1/1000), p38 (8690P 1/1000), p‐p38 (4511P 1/1000), ERK (4695P 1/1000), p‐ERK (4370P 1/2000) was sourced from Cell Signaling Technology.

### 2.9 Rat prolactinoma model

In this study, rat pituitary tumors were induced in 4‐week‐old female F344 rats by subcutaneously implanting 1 cm silastic capsules containing 10 mg of 17‐β estradiol. Prolactinomas were induced using 17β‐estradiol for 5 weeks, following a previously described method. All experimental procedures were conducted by the guidelines and regulations set by the Animal Use and Care Committee of Beijing Tiantan Hospital. Before intrapituitary injection, the presence of prolactinomas was confirmed through 7.0‐T magnetic resonance imaging (MRI). The rats were anesthetized, and each bilateral tumor was stereotactically injected with either adenovirus vector control, WT‐PBK, or PBK‐T9F (1 μL). Another MRI was performed 2 weeks later.

### 2.10 Xenograft experiments

The study received approval from the Ethics Committee of Beijing Tiantan Hospital. A total of twenty male BALB/c nude mice, aged 6 weeks, were randomly assigned to two groups. Each mouse in both groups received a subcutaneous injection of 3 × 10^6^ GH3 cells transfected with either PBK or PBK‐T9F in a serum‐free medium, specifically in the right axilla. After 4 weeks following the injection, the mice were euthanized, and the tumor volumes and weights were measured.

### 2.11 Immunohistochemistry techniques

Before conducting immunohistochemistry (IHC), the TMA slides underwent staining with hematoxylin and eosin and were assessed for both quality and tumor content. The TMAs were then processed using the Leica BOND‐III, an automated, random, continuous‐access slide‐staining system located in Nussloch, Germany. This system allowed for the simultaneous execution of multiple IHC assays. To detect the primary antibody, the Bond Polymer Refine Detection system from Leica Biosystems was employed. The resulting immunostained slides were subsequently examined for expression using the Aperio AT2 digital scanner, also from Leica Biosystems. Staining intensity was categorized into four distinct levels: 0, negative; 1+, weak; 2+, moderate; and 3+, strong. The percentage of immunostaining was recorded and H‐scores were calculated as:

$$H-\text{score}=\left(\%\text{cells}\;1+\right)+2*\left(\%\text{cells}\;2+\right)+3*\left(\%\text{cells}\;3+\right)$$

The maximum H‐score was 300, corresponding to 100% of cells stained with strong intensity.

### 2.12 Patients

This study utilized a total of 81 functional pituitary neuroendocrine tumors (PitNETs) obtained from tumor resections conducted at Beijing Tiantan Hospital, Capital Medical University, during the period from 2020 to 2022. Invasive pituitary adenomas were classified as Knosp grade 3 and 4, as determined by magnetic resonance imaging (MRI). The research protocol received approval from the Ethics Committee of Beijing Tiantan Hospital, and informed consent was obtained from all participating patients. Detailed clinical characteristics of the patients can be found in Table 1.

**TABLE 1:** Correlation between pPBK‐T9 expression and clinical characteristics.

Characteristic	High	Low	*p*
*n*	47	34	
Age, *n* (%)			1.000
≤58	35 (43.2%)	26 (32.1%)	
>58	12 (14.8%)	8 (9.9%)	
Sex, *n* (%)			1.000
Female	20 (24.7%)	14 (17.3%)	
Male	27 (33.3%)	20 (24.7%)	
Pathology, *n* (%)			0.334
GH	18 (22.2%)	9 (11.1%)	
PRL	21 (25.9%)	15 (18.5%)	
ACTH	8 (9.9%)	10 (12.3%)	
Invasion of bone, *n* (%)			0.672
No	31 (38.3%)	20 (24.7%)	
Yes	16 (19.8%)	14 (17.3%)	
Volume classification, *n* (%)			0.637
Microadenoma	3 (3.7%)	4 (4.9%)	
Macroadenoma	33 (40.7%)	21 (25.9%)	
Giant adenoma	11 (13.6%)	9 (11.1%)	
ki67, *n* (%)			0.408
<3	14 (17.3%)	14 (17.3%)	
≥3	33 (40.7%)	20 (24.7%)	
Primary/recurrent, *n* (%)			1.000
Primary	38 (46.9%)	28 (34.6%)	
Recurrent	9 (11.1%)	6 (7.4%)	
Knosp, *n* (%)			0.001*
0–2	15 (18.5%)	24 (29.6%)	
3–4	32 (39.5%)	10 (12.3%)	

*Showed the results are statistically different.

### 2.13 Tumor samples and tissue microarray construction

Hematoxylin and eosin staining was performed on formalin‐fixed paraffin‐embedded tissue slices. From paraffin‐embedded tissue blocks, three sections of core biopsies measuring 2.0 mm in diameter were chosen and transferred to tissue microarrays (TMAs) using a Minicore Tissue Arrayer (Mitogen, UK). The TMAs were then sliced into 4‐μm sections using a continuous microtome. To ensure anonymity and minimize loss of antigenicity, the samples were randomly arranged and anonymized on TMA slides. The microarray slices were processed within a week to prevent further loss of antigenicity.

### 2.14 Statistical analysis

The statistical analyses were conducted using GraphPad Prism 8.00 statistical software. The experimental data were presented as the mean ± SD (standard error of measurement) of three or more independent experiments, as specified in the corresponding figure legends and methods. The normality of the data distribution was assessed using the Shapiro–Wilk test. The comparisons between the two groups were performed using Student's *t*‐test or Mann–Whitney U test (nonparametric). Additionally, differences among the three groups were assessed using one‐way ANOVA or Kruskal–Wallis test (nonparametric). *p* < 0.05 was considered to indicate a significant difference.

## 3 RESULTS

### 3.1 CDK5 promotes cell proliferation and EMT progression in GH3 cells

**FIGURE 1:**
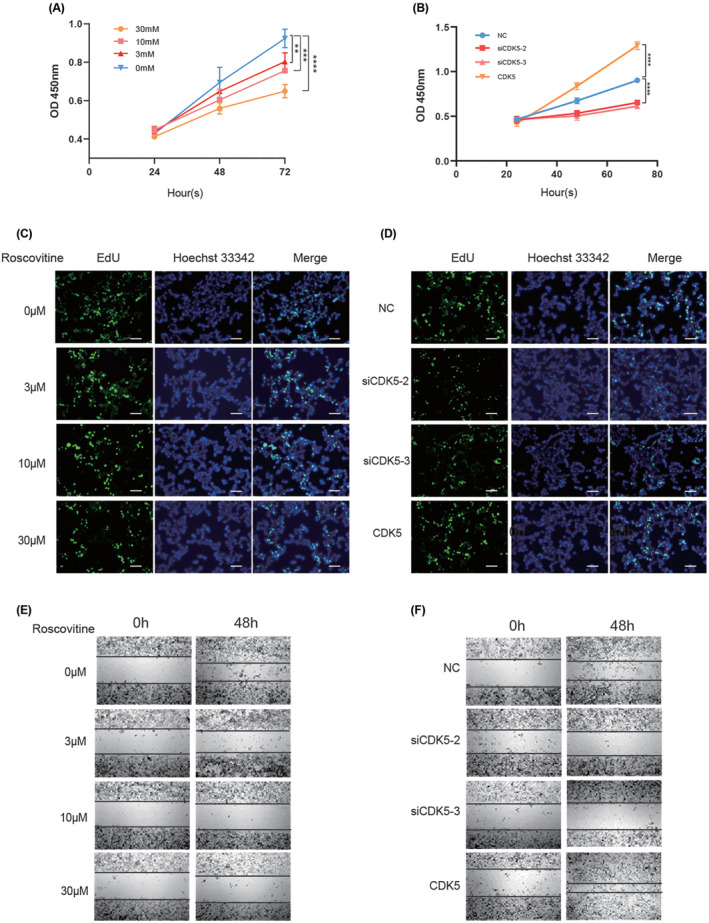
CDK5 promotes cell proliferation and EMT progression in GH3 cells. (A, B) Growth curves of GH3 cells treated with roscovitine and transfected with siCDK5‐2, siCDK5‐3, and CDK5. (C, D) *EdU* assay for detecting cell proliferation. E and F Wound healing assay for detecting cell invasion. **, *p* < 0.01; ***, *p* < 0.001; ****, *p* < 0.0001. The bar represents the mean ± SD.

**FIGURE 2:**
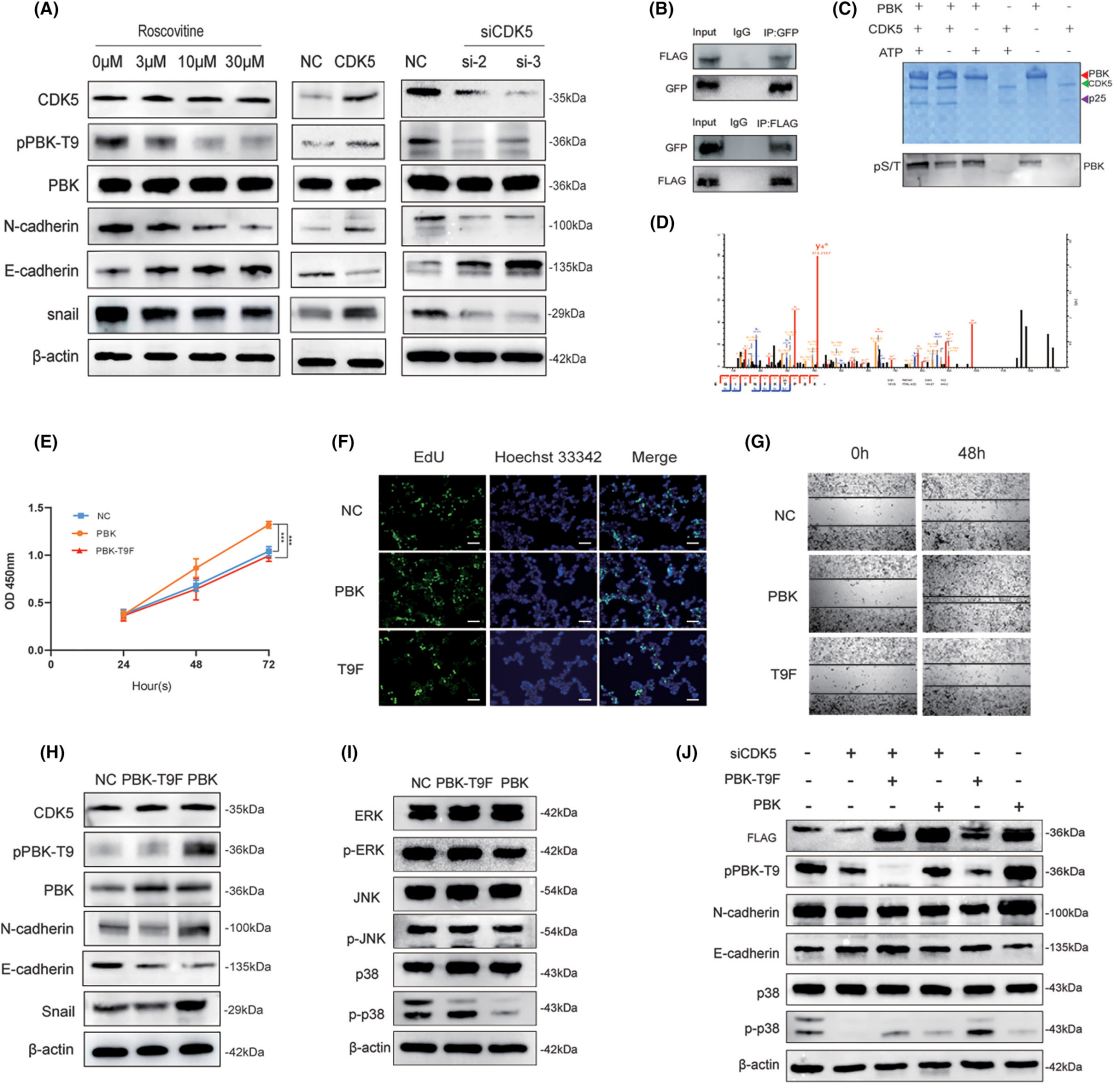
Phosphorylation of PBK at Thr9 by CDK5 promotes cell proliferation and EMT progression. (A) GH3 cells treated with roscovitine and transfected with siCDK5‐2, siCDK5‐3, and CDK5. Western blot analysis of pPBK‐T9, PBK, CDK5, and EMT expression. (B) Co‐IP to detect the interaction between CDK5 and PBK. (C) I*n* vitro *kinase* assay was performed to test whether CDK5 phosphorylates PBK at Thr9. (D) The phosphorylation site of PBK at Thr9 was analyzed by mass spectrometry. (E) Growth curves of GH3 cells transfected with PBK and PBK‐T9F. (F) *EdU* assay for detecting GH3 cells transfected with PBK and PBK‐T9F. (G) Wound healing assay for detecting cell invasion. (H) Western blot analysis of pPBK‐T9, PBK, CDK5, and EMT expression after GH3 cells were transfected with PBK and PBK‐T9F. (I) GH3 cells were transfected with PBK and PBK‐T9F, and the expression of MAPK *signaling pathway‐related proteins was determined*. (J) Western blot analysis was performed to test whether PBK reversed the effect of siCDK5 on EMT progression. β‐Actin served as a loading control. **, *p* < 0.01; ***, *p* < 0.001; ****, *p* < 0.0001. The bar represents the mean ± SD.

To determine the influence of CDK5 on cell proliferation and EMT, We cultured GH3 cells with either treatment with roscovitine (a CDK5 inhibitor) at different concentrations. We detected cell proliferation using CCK‐8 and EdU cell proliferation assays. As shown in Figure 1A, cell proliferation gradually decreased as the roscovitine concentration increased, and cell activity was significantly inhibited in GH3 cells treated with 30 μm roscovitine. Likewise, the above results were further verified by the EdU assay (Figure 1C). Next, we transfected GH3 cells with CDK5 and short interfering RNA (siRNA) targeting CDK5 mRNA. Compared with the NC group, the CDK5 group cell proliferation was significantly increased (Figure 1B). The proliferation of GH3 cells was inhibited. Similar results were obtained in the EdU assay after CDK5 knockdown (Figure 1D). Moreover, CDK5 significantly attenuated the invasion of GH3 cells, according to wound healing assay results (Figure 1E,F). We then investigated by WB whether EMT‐associated markers are affected by CDK5 in GH3 cells (Figure 2A). We found that EMT progression was significantly inhibited after treatment with the CDK5 inhibitor roscovitine. The levels of N‐cadherin and Snail gradually decreased in a dose‐dependent manner after roscovitine intervention, and the protein level of E‐cadherin increased, but the CDK5 and PBK protein levels remained unchanged. After CDK5 knockdown, it appears that EMT has attenuated overall based on the pattern of changes in EMT markers. EMT activation was observed only in CDK5‐overexpressing cells. Collectively, these data indicated that CDK5 contributed to cell proliferation and EMT progression in GH3 cells.

### 3.2 Phosphorylation of PBK at Thr9 by CDK5 promotes cell proliferation and EMT progression

*Through* consulting the *literature*, we were particularly *interested in PBK, which* is overexpressed *in* several human *malignancies*.^20^, ^21^, ^22^, ^23^, ^24^ By analyzing the amino acid sequence of PBK, we found that PBK at Thr9 may be the potential phosphorylation site of CDK5. To further verify that CDK5 phosphorylates PBK‐T9, HEK293 cells were transfected with GFP‐CDK5 and FLAG‐PBK. Beads with anti‐FLAG or GFP antibodies were used for co‐IP, and WB was used to check the co‐IP results. Co‐IP results show the interaction between PBK and CDK5 (Figure 2B). To further verify phosphorylation, an in vitro kinase assay was performed. We incubated purified CDK5 and PBK proteins with ATP in vitro kinase assays, and the data indicated that CDK5 phosphorylates PBK in vitro (Figure 2C). PBK autophosphorylation is dependent on the presence of ATP in vitro. Whole lanes were analyzed by mass spectrometry to identify the purified proteins. The results indicated that Thr9 was phosphorylated (Figure 2D). Moreover, WB results showed that the pPBK‐T9 and CDK5 protein expression trends exhibited the same trend, with both CDK5 being upregulated or downregulated (Figure 2A).

To understand the importance of pPBK‐T9 for proliferation and EMT, we constructed a PBK inactivated mutant (PBK‐T9F) and a TOPK overexpression vector (PBK‐WT), and we transfected the above two plasmids into GH3 cells. Compared with the PBK‐T9F group, PBK remarkably facilitated cell proliferation in CCK‐8 and EdU cell proliferation assays (Figure 2E,F). The wound healing assay results show that PBK promotes the invasion of GH3 cells (Figure 2G). WB results showed that PBK‐T9 phosphorylation promoted EMT progression (Figure 2H). Many signal transduction pathways, including MAPK, PI3K/AKT, and mTOR, are associated with PBK, suggesting that PBK might play a role in autophagy regulation and EMT.^25^, ^26^ To further determine the activation levels of their downstream signaling pathways, we measured the relative expression of MAPK‐related proteins and phospho‐proteins. After changes in PBK phosphorylation levels, WB suggested that ERK, p‐ERK, JNK, and p‐JNK had no significant change. However, the phosphorylation of p38 significantly decreased as pPBK‐T9 increased, and total p38 remained unchanged (Figure 2I). As shown in Figure 2J, WB results showed that siCDK5 and PBK‐T9F reduced the level of PBK‐T9 phosphorylation compared with the PBK group and suppressed the progression of EMT, but PBK reversed these inhibitory effects and suppressed the promoting effect of PBK‐T9F on p‐p38. Above all, our data suggested that CDK5 could promote the phosphorylation of PBK‐T9 through the p38 MAPK signaling pathway to promote cell proliferation and EMT progression.

### 3.3 Phosphorylation of PBK at Thr9 by CDK5 enhances the stability of PBK

**FIGURE 3:**
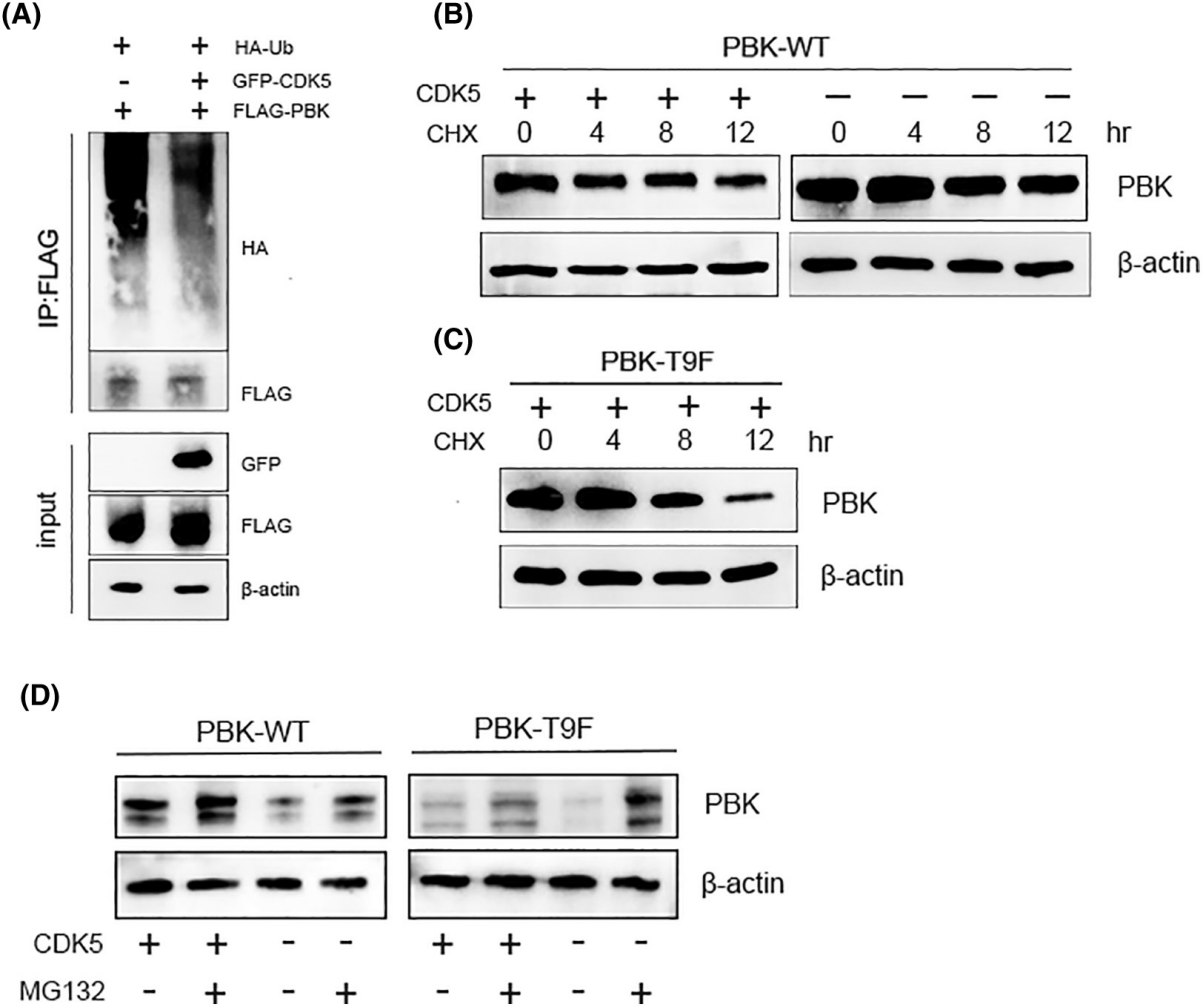
Phosphorylation of PBK at Thr9 by CDK5 enhances the stability of PBK. (A) HEK293T cells were transfected with the indicated plasmids, and cell extracts were subjected to IP with an anti‐FLAG antibody. Ubiquitinated PBK was detected by immunoblotting. (B, C) HEK293T cells were transfected with the indicated plasmids and then treated with CHX (100 μg/mL) to prevent new protein synthesis. The time‐dependent stability of PBK was detected by Western blot. (D) Validation of proteasome degradation pathways. β‐Actin served as a loading control.

We next investigated whether the phosphorylation of PBK at Thr9 by CDK5 affects the stability of PBK. First, GFP‐CDK5, FLAG‐PBK, and HA‐ubiquitin were transfected into HEK293T cells. After forty‐eight hours, the cell extracts from each group were subjected to immunoprecipitation using anti‐FLAG, and the presence of HA‐ubiquitin was detected through Western blot analysis. The obtained results demonstrated a significant reduction in the ubiquitination level of PBK due to the overexpression of CDK5, as compared to the control group (Figure 3A). To further validate these findings, the half‐life of PBK was investigated. HEK293T cells were transiently transfected with PBK‐WT or PBK‐T9F, along with CDK5, and subsequently treated with CHX to evaluate the stability of PBK at different time intervals. The results showed that the half‐life of PBK in *CDK5*‐overexpressing cells was much longer than that in control cells. PBK‐T9F had a shorter half‐life than PBK‐WT (Figure 3B,C). We then used CHX and a proteasome‐specific inhibitor MG132 to investigate whether CDK5 increases protein accumulation by phosphorylating T9 of PBK. As shown in the results (Figure 3D), MG132 significantly reduced the degradation of PBK protein, indicating that CDK5 does upregulate PBK by inhibiting the proteasome pathway. In summary, these results elucidate that phosphorylation of PBK‐T9 by CDK5 enhances the stability of PBK.

### 3.4 Phosphorylation of PBK at Thr9 by CDK5 promotes prolactinomas growth in vivo

**FIGURE 4:**
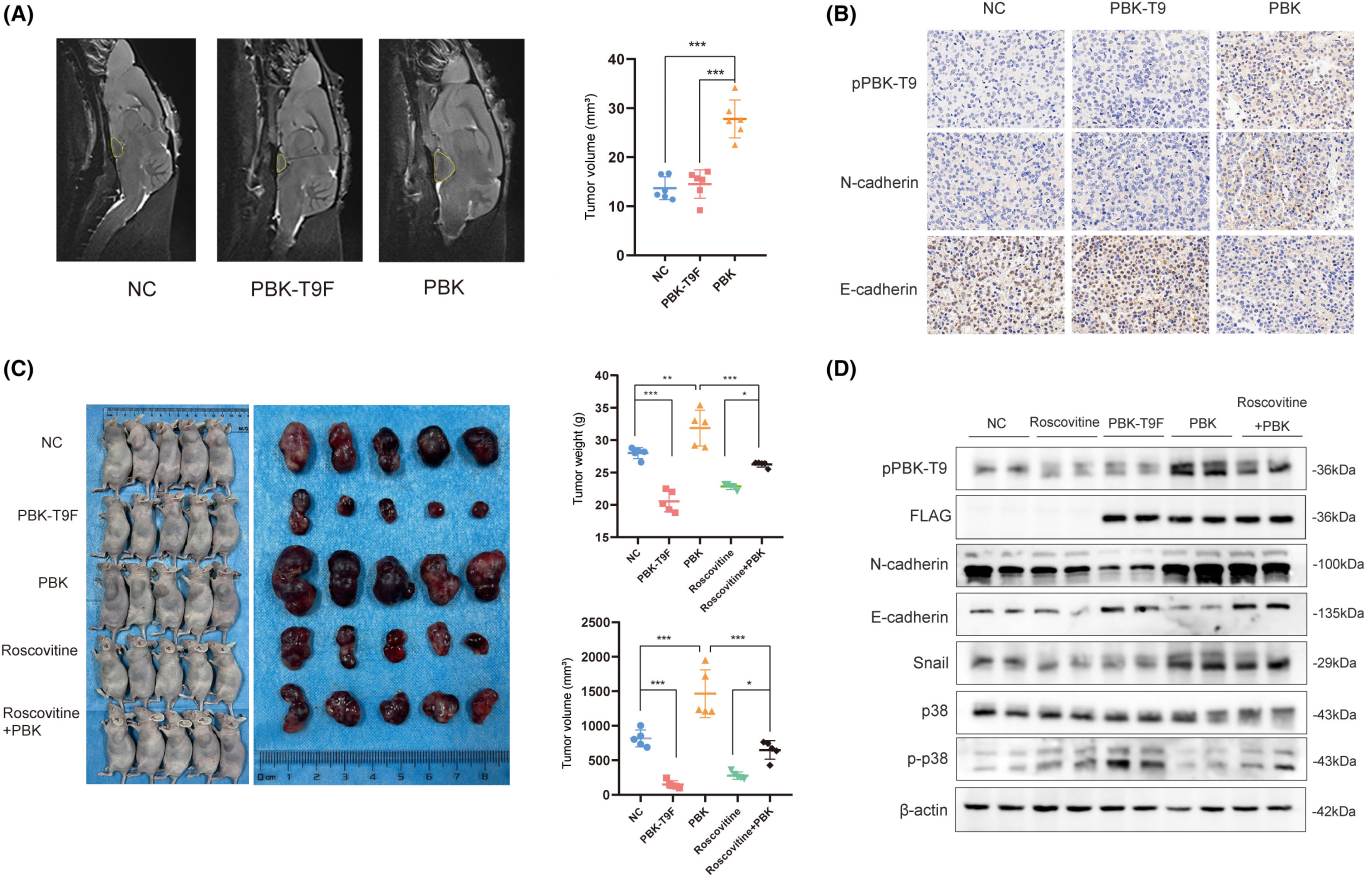
Phosphorylation of PBK at Thr9 by CDK5 promotes prolactinoma growth in vivo. (A) Prolactinomas in F344 rats were induced by E2 administration. MRI shows lesions located on the seller's floor (left). The pituitary tumor volume was measured by MRI (right). (B) IHC analysis of pPBK‐T9, E‐cadherin, and N‐cadherin expression in tumor sections from prolactinomas in F344 cells. Magnification, 400×. (C) Tumors dissected from the NC, PBK, PBK‐T9F, roscovitine, and roscovitine+PBK groups are shown (left). Growth curve of tumor size and average tumor weight (right). (D) Western blot analysis of the expression of the indicated markers in tumor sections from mice in the NC, PBK, PBK‐T9F, roscovitine, and roscovitine+PBK groups. β‐Actin served as a loading control. *, *p* < 0.05; **, *p* < 0.01; ***, *p* < 0.001. The bar represents the mean ± SD.

We verified the above in vitro data in vivo by inducing a rat prolactinoma model. F334 rat prolactinomas were first induced by 17*β*‐estradiol for 6 weeks. Then, the rat prolactinomas were stereotactically injected with PBK or PBK‐T9F *adeno‐associated virus (AAV)*. After 2 weeks, the mean tumor volume was significantly increased from the PBK group to the PBK‐T9F group, validating that high pPBK‐T9 promoted prolactinoma growth in vivo (Figure 4A). The immunohistochemistry results show that PBK drives EMT progression in rat prolactinomas (Figure 4B). These results suggested that high pPBK‐T9 promoted prolactinoma growth in vivo.

We then verified the above results in a xenograft tumor‐formation assay. We compared the ability of PBK‐WT and PBK‐T9F *stable* cells to form tumors in athymic nude mice. PBK‐WT or PBK‐T9F cells (2 × 10^6^) were injected subcutaneously into the left flank of athymic BALB/c mice. Thirty days after transplantation, roscovitine was injected intraperitoneally into the mice, followed by two weekly cycles of intraperitoneal administration of drugs. Compared with those from the PBK‐T9F group and control group, tumors from the PBK‐WT group were much larger, and roscovitine treatment also led to a tumor volume and a tumor weight that were dramatically lower than those induced by PBK‐WT. Roscovitine reversed the tumor‐promoting effect of PBK (Figure 4C). WB analysis of the tumor samples from the two groups of mice showed that pPBK‐T9 was more highly expressed in PBK‐WT tumors than in NC or PBK‐Y9F tumors (Figure 4D), but p‐p38 protein expression was decreased in PBK‐WT tumors. Meanwhile, roscovitine reverses the progression by which PBK promotes EMT and completely reverses the inhibitory effect of PBK on p‐p38. These data showed that the tumorigenic properties of PBK‐WT cells were significantly increased, suggesting that CDK5 phosphorylates PBK‐T9 via the p38 MAPK signaling pathway and promotes prolactinoma growth in vivo.

### 3.5 pPBK‐T9 is highly expressed in invasive PitNETs

**FIGURE 5:**
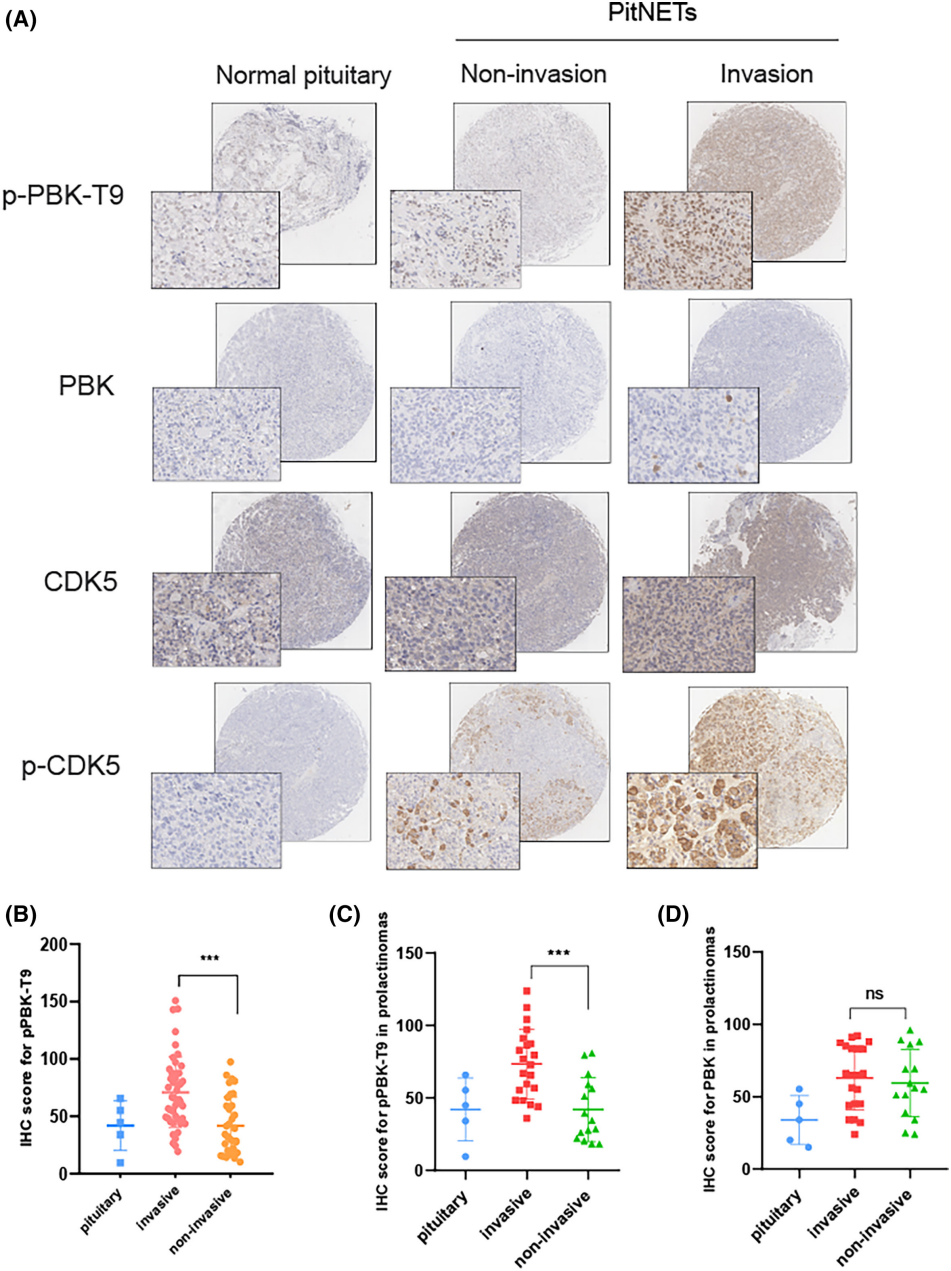
pPBK‐T9 is highly expressed in invasive PitNETs. (A) IHC analysis of pPBK‐T9, PBK, CDK5, and *p*‐CDK5 expression in PitNET tissue samples. Magnification, 400×. (B) IHC score of pPBK‐T9 in different Knop grade PitNETs. (C, D) IHC score of pPBK‐T9 and PBK in prolactinomas. ***, *p* < 0.001. The bar represents the mean ± SD.

We constructed 81 functional PitNETs and 6 normal pituitary tissue samples. Based on the *Knosp* classification, we classified a total of 81 cases as invasive (*n* = 39) and noninvasive (*n* = 42). We analyzed the expression of pPBK‐T9 in PitNET tissues by IHC. As a result, higher pPBK‐T9 and pCDK5 expression was observed in invasive PitNETs than in noninvasive PitNETs (Figure 5A), but total PBK and CDK5 expression was not significantly different. The staining results were scored according to the H‐Score system and then counted (Figure 5B). *As* expected, the data showed that the IHC score of pPBK‐T9 was higher in invasive PitNETs than in noninvasive PitNETs. There were 30 prolactinomas in 81 functional PitNETs. We evaluated the expression of pPBK‐T9 and PBK in prolactinomas by IHC (Figure 5C,D). pPBK‐T9 was higher in invasive prolactinomas than in noninvasive prolactinomas, and there was no significant difference in PBK expression, suggesting an oncogenic role of pPBK‐T9 in PitNETs. To study the correlation of pPBK‐T9 expression with clinical characteristics in PitNETs. The patients were divided into high and low expression of pPBK‐T9 by the IHC score of pPBK‐T9. Patient characteristics are shown in Table 1. Clinical characteristic analysis showed that pPBK‐T9 expression was correlated with Knosp grade but not with other features (Table 1). For further validation, the correlation between pPBK‐T9 expression and tumor invasion was assessed, and the patients were divided into noninvasive PitNETs and invasive PitNETs by MRI. Logistic regression analysis was performed to determine the factors associated with invasive PitNETs (Table 2). In the univariate analysis, bone invasion, volume classification, and pPBK‐T9 expression were found to be factors associated with invasive PitNETs. In the multivariate survival analyses, pPBK‐T9 expression was identified as a significant prognostic factor. Above all, pPBK‐T9 is highly expressed in invasive PitNETs and was significantly and independently associated with invasion.

**TABLE 2:** Univariate and multivariate Logistic regression analysis.

Characteristics	Univariate analysis		Multivariate analysis	
	OR (95% CI)	*p*	OR (95% CI)	*p*
Age (≥45 years vs. <45 years)	2.75 (0.93–8.11)	0.067		
Sex (Female vs. Male)	1.39 (0.58–3.38)	0.463		
Ki67 (High vs. Low)	1.39 (0.56–3.49)	0.478		
Pathology (ACTH /PRL vs. GH)	1.87 (1.01–3.49)	0.048		
Primary/recurrent (Primary vs. recurrent)	2.13 (0.65–6.89)	0.209		
Invasion of bone (Yes vs. No)	4.26 (1.59–11.4)	0.004*	1.24 (0.24–6.37)	0.799
Volume classification (Macro vs. Micro)	9.09 (2.63–31.4)	<0.001*	2.38 (0.33–17.3)	0.390
pPBK‐T9 expression (High vs. Low)	5.12 (1.96–13.3)	0.001*	11.8 (2.59–53.4)	0.001*

*Showed the results are statistically different.

## 4 DISCUSSION

Invasive PitNETs often invade surrounding anatomical structures that are rapidly growing and large and have a poor prognosis. Invasive PitNETs are prone to recurrence after surgical resection and are difficult to cure, and their residual tumor may even be aggravated.^27^, ^28^, ^29^, ^30^ However, the pathogenesis of pituitary tumors has not been clearly defined. Our study shows that the phosphorylation level of PBK‐T9 is highly expressed on invasive PitNET tissues, and we clarified that invasiveness was significantly correlated with the phosphorylation level of PBK‐T9 by univariate and multivariate analyses. Similarly, we confirmed that increased levels of pPBK‐T9 could promote EMT progression in GH3 cells.

Accumulating evidence supports the role of PBK in mitosis and cell‐cycle progression of mitotically active cells. Cyclins and their catalytic partners, cyclin‐dependent kinases (CDKs), control the transition between different phases of the cell cycle.^31^, ^32^ PBK promotes mitotic advance via the cdk1/cyclin B1‐dependent phosphorylation of PRC1, and PBK induces the phosphorylation of PRC1 at T481 only when cdk1/cyclinB1 exists simultaneously^33^; however, there is no clear relationship between PBK and other CDKs. A great deal has been done to elucidate the mechanisms of CDK5 phosphorylation. We confirmed that CDK5 phosphorylated KDR (Ser‐229) promotes tumor development.^19^ In preliminary work, we collected 6 Pit‐1‐positive PitNETs to perform single‐cell RNA‐seq analyses (data not yet published). We noted malignant transformation subpopulations, including PBK, TOP2A, PTTG1, and MKI67. CDK5, a serine/threonine kinase, exhibits stringent sequence prerequisites for its phosphorylation substrate, exclusively targeting serine or threonine residues harboring conserved S/TPXX (K/R/H) motifs. The Thr9 of PBK exactly satisfies the phosphorylation site requirements of CDK5. In the present study, the phosphorylation level of PBK‐T9 was modified by regulating CDK5 protein expression, and CDK5 phosphorylation of PBK‐T9 was confirmed by in vitro kinase assays and co‐IP. Several studies have reported that the nuclear translocation of CDK5 inhibits the proliferation and tumorigenicity of cancer cells, and CDK5 inhibitors (roscovitine) interfere with the nuclear translocation of CDK5 from the cytoplasm during the phosphorylation of p53 by CDK5.^34^, ^35^, ^36^

PBK is a MAPKK‐like protein that may be involved in the ERK, JNK, and p38 MAPK signaling pathways in a cell type‐dependent manner.^36^ Based on the literature, one identified TOPK substrate and widely used mitotic marker, p38 MAPK, attracted our attention.^15^ The contribution of p38 MAPK phosphorylation by PBK to cell survival has been documented in previous studies.^36^ Additionally, it has been suggested that increased expression of PBK may promote tumor growth by facilitating p38 activation and aiding cells in overcoming DNA damage.^37^ However, the regulatory role of PBK phosphorylation on Tyr or Thr residues within the MAPK signaling pathway has not yet been reported. We found that upregulating the level of pPBK‐T9 significantly suppressed the phosphorylation of *p38*, but the expression of total *p38* remained unchanged regardless of pPBK‐T9 expression. However, a considerable amount of literature has reported that *p‐p38* pathways are upregulated and tumor cell proliferation, migration, and invasion are induced.^37^, ^38^, ^39^, ^40^ However, our data showed that CDK5 could phosphorylate PBK‐T9 to suppress p‐p38 expression, promoting cell proliferation and EMT progression. We suppose that positive or negative regulation of p‐p38 may enhance tumor development. It has been reported that TNFα‐induced apoptosis is dependent on the activation of p38 in neutrophils.^41^ Prx6 regulates EMT signaling by reducing p38 phosphorylation in colon cancer cells.^42^ *The* above *results* confirmed *our conjecture*. As the function of PBK has been revealed, a variety of PBK small molecule inhibitors have been reported: HI‐PBK‐032, OTS514, OTS964, and ADA‐07 have a strong inhibitory effect on PBK in a variety of tumors. Unfortunately, no drug can specifically inhibit the phosphorylation of PBK‐T9.

**FIGURE 6:**
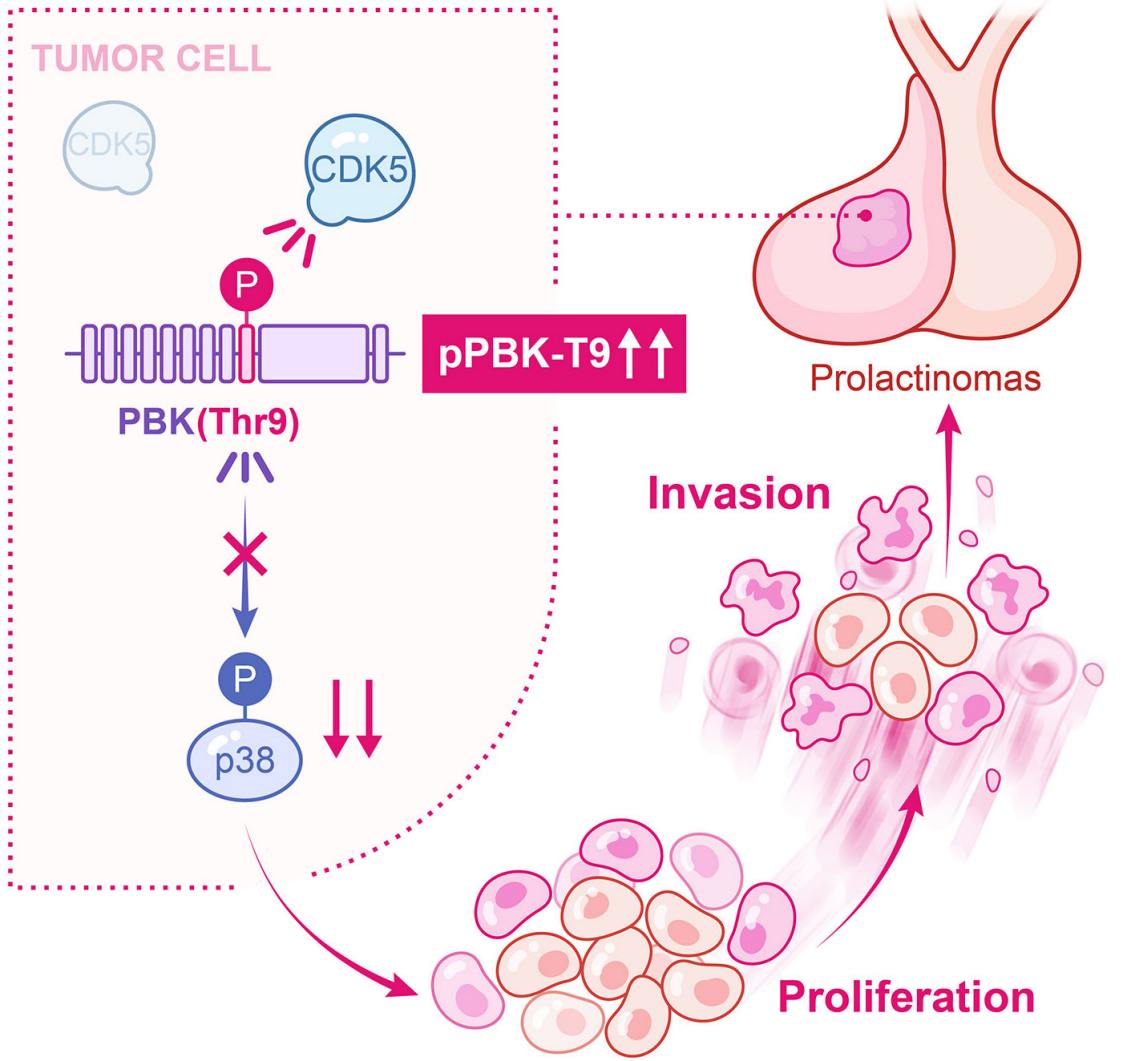
Schematic representation showing the proposed mechanisms through which the Phosphorylation of PBK at Thr9 by CDK5 promotes the prolactinoma progression.

In the current study, we first report that CDK5 phosphorylates PBK at Thr9 and promotes tumorigenesis. pPBK‐T9 is highly expressed in invasive PitNETs. Additionally, our findings showed that CDK5 inhibitors could directly or indirectly block proliferation and EMT in GH3 cells. Most importantly, we revealed that this signaling axis in PitNETs, CDK5/PBK/p38 MAPK, promoted the tumorigenesis of PitNETs in vitro, highlighting a new molecular mechanism in PitNETs (Figure 6).

## AUTHOR CONTRIBUTIONS

WX and YZ worked on the conception and designed the research. CL and was involved in the collection and analysis of patients' clinical data. JG and QF performed the experiments. QF and DN were dedicated to data analysis, interpretation, drafting, and revising the manuscript. All authors read and approved the final manuscript.

## CONFLICT OF INTEREST STATEMENT

The authors declare that they have no competing interests.

## CONSENT FOR PUBLICATION

Not applicable.

## Data Availability

The datasets used and/or analyzed during the current study are available from the corresponding author upon reasonable request.
